# Progressively Increased Range of Motion Confers Similar Strength Improvements but Not Bar Kinematics as Full Range of Motion Bench Press

**DOI:** 10.3390/jfmk11010072

**Published:** 2026-02-11

**Authors:** Michael J. Landram, Patrick Manturi, Mark Zipagan, Emily E. Gerstle

**Affiliations:** Department of Health and Human Performance, The University of Scranton, 800 Linden St., Scranton, PA 18510, USAemily.gerstle@scranton.edu (E.E.G.)

**Keywords:** bench press, overload, range of motion, partial, bar kinematics

## Abstract

**Background**: Full versus partial range of motion (ROM) bench press (BP) training has only been investigated at submaximal loads with discrete joint angles during training. The aim of this study was to compare the effects of a 4-week supramaximal progressive partial ROM (pROM) BP program to a traditional submaximal full range of motion (fROM) program on 1-RM strength and bar kinematics. **Methods**: Sixteen resistance-trained males (22.2 ± 1.4 years, 180.1 ± 6.3 cm, 88.5 ± 8.6 kgs, 1RM ≥ 1.25× body mass, 6 years’ experience) were randomized into pROM (n = 7) or fROM (n = 9). The pROM group performed BP at 105% 1RM using Bench Blokz to decrease the distance from the bar to the sternum by 1″ increments each week (5″ to 2″). The fROM group followed a strength oriented linear periodization model (80–87.5% 1RM). Both 1RM strength and 3D kinematics were assessed pre- and post-intervention using a 2 × 2 (Group × Time) ANOVA with Bonferroni corrected pairwise comparisons. **Results**: Both groups significantly increased 1RM strength (F = 45.82, *p* < 0.001), with no significant differences between groups. Pairwise comparisons revealed that only the fROM group experienced significant increases in 1st peak velocity (*p* = 0.023), eccentric velocity (*p* = 0.009), mean concentric force (*p* = 0.04) and quartile 2 mean concentric force (*p* = 0.01). **Conclusions**: Supramaximal pROM training is an effective strategy for increasing 1RM strength in experienced lifters, yielding results comparable to traditional fROM training over the course of a 4-week strength block. However, there are notable changes in bar kinematics surrounding the eccentric-concentric phase change that were only observed after fROM training.

## 1. Introduction

The bench press (BP) is one of the most performed exercises in strength and conditioning. It is used to develop strength and hypertrophy upper body musculature [1], assess upper body strength and endurance [2], and rehabilitate from injury [3]. A primary advantage of the barbell BP over other horizontal pushing movements, such as pushups, is the ability to progressively load resistance beyond an individual’s body weight [4]. As lifters advance to the point where the resistance of a push up is insufficient and extra loads are necessary for progression, a variety of biomechanical and physiological issues arise when working through a full range of motion (fROM) [5].

One such limitation of fROM BP training is that lifters encounter a sticking point or sticking period where concentric bar velocity decreases from its first peak velocity to the first minimum velocity [6,7]. This appears to happen for a variety of reasons that result in technique breakdown leading to deviations in bar kinematics from an ideal path [8,9]. To address technique inconsistencies near the sticking period, lifters may include partial range of motion (pROM) lifting in their training. The idea behind pROM is that an individual is able to overload, through either volume or intensity, the area in question [5,9,10]. Indeed, working from a specific pROM or multiple pROMs is frequently used when training BP in athletic and rehabilitative populations. In a survey by Swinton et al. [11], a majority of competitive powerlifters (57.1%) reported using board presses in their training. The board press is a way to limit ROM by placing stacked wooden boards on the individual’s chest, decreasing the distance the bar can travel. Generally, the pROM would be changed throughout the training block to either address a sticking period or carry over to fROM performance. Similarly, athletes may utilize a towel roll or bar padding to limit ROM to overload the BP movement, avoid overuse injury, or rehabilitate an injured joint [3,4,12].

While no study has investigated progressively increasing pROM towards fROM, many have compared training at specific fixed pROMs to fROM. Clark et al. [9] were the closest to report on what could be considered progressive pROM BP training. In their study, six males with resistance training backgrounds performed four sessions of variable ROM bench press training where they would perform a 6-repetition maximum (RM) set at ¼, ½, ¾ lockout in a smith machine over a 4-week period leading to 6RM testing. However, this study did not have pre-intervention testing to compare strength changes and was only able to describe differences in loads between pROMs, which increased as the pROM decreased. As well, while the authors reported greater statistical power than previous studies examining pROM testing [5], there is still room for improvement.

Massey and colleagues’ [13] methodology contained groups that would most closely resemble the way powerlifters have reported using pROM in their training by combining both fROM and pROM across BP sets. They compared groups that would perform either fROM BP for all sets or pROM 2–5″ from the lockout position for all sets. They reported significant improvements to 1RM in all groups with no difference between each group. However, due to the untrained status of the participants, the novelty of engaging in a resistance training program limits the generalizability of these results.

In 2019, Martínez-Cava and colleagues [14] evaluated ROM and sticking region effects on the BP load–velocity relationship. Here, the authors describe these relationships after testing 1RM strength at fROM, ⅓ pROM and ⅔ pROM. Similar to previous reports [9], loads lifted in this study increased as the pROM decreased. During the fROM max test researchers observed two velocity peaks with the sticking period being identified as the minimum velocity between the two. Neither this decreased velocity to a minimum after the 1st peak, nor a 2nd peak velocity was observed in the ⅓ or ⅔ pROM groups. Interestingly, participants testing fROM 1RM experienced this sticking period of minimum velocity before the ⅓ or ⅔ ROM, yet during ⅔ pROM 1RM testing, 54.5% of individuals began the BP movement before this sticking period and experienced no similar slowdown before completing the lift. This suggests that there may be alterations in biomechanics before this sticking region is encountered, which would lead to the minimum velocity drop. This study utilized trained individuals with a 1RM BP max ≥ 0.8× body mass who have trained BP over the past year. Although the authors used extensive familiarization with the pROM groups, there was no training to measure longitudinal changes in performance and using a smith machine to test BP makes transferring bar kinematics to free weights difficult. However, in 2022, Martínez-Cava et al. [15] recruited resistance trained participants into four groups that would undergo velocity-based resistance smith machine BP training, and participants completed 10 weeks of either fROM, ⅓ pROM, or ⅔ pROM or stopped training entirely. All training groups utilized the same relative loads 60–80% 1RM. Before and following their training, all participants tested 1RM strength in all ROM conditions. Martínez-Cava reported that fROM had the greatest carryover to pROM 1RM testing, and pROM offered smaller improvements as the pROM decreased. However, velocity-based training is a relatively new construct that requires additional equipment to measure during training, thus limiting its application. Additionally, as noted by others [5,9,10] a specific benefit of utilizing pROM is the ability to overload the limited ROM; therefore, the differences observed in this study may be the result of undertraining the pROM groups compared to fROM.

Taken together, while previous studies have examined single or multiple pROM at submaximal intensities, no study has supramaximally loaded a pROM or progressively increased the pROM and compared it to fROM over the course of a training block. Therefore, the purpose of this study was to compare the effects of a 4-week supramaximal progressively increased pROM to a traditional submaximal fROM BP training program on strength outcomes and bar kinematics in well-trained males.

## 2. Materials and Methods

### 2.1. Subjects

Sixteen resistance-trained college-aged males volunteered to participate in this study (Table 1). Participants were free of upper body injury, had been completing at least two horizontal pressing training sessions per week over the past year, had not used partial range of motion (pROM) training in the past year, and had a BP 1RM ≥ 1.25× body mass. Based on prior studies [13,15], a sample size of 10 participants would be required to estimate direct differences in 1RM with a power of 80% and a significance α of 0.05 using GPower 3.1. The study was completed in compliance with the Declaration of Helsinki and was approved by the Institutional Review Board of the University of Scranton (24-16B). Written informed consent was obtained from all participants prior to enrolling in the study.

### 2.2. Study Design

Participants were randomly assigned to complete a 6-week study. The first and last weeks would test 1RM with the intervening 4 weeks consisting of either fROM BP following a linear periodization strength block model (80–87.5% 1RM) or a progressively increasing pROM BP with supramaximal intensity (105% 1RM). Both groups trained BP under the supervision of investigators for 2 sessions each week with training sessions separated by at least 72 h. Participants were instructed to abstain from stimulants and alcohol for 24 h before 1RM testing and training sessions.

### 2.3. Anthropometric and Body Composition Assessment

Participants underwent a DXA (GE Healthcare, Madison, WI, USA) scan to analyze body composition. Additionally, to ensure no musculoskeletal deviations were present that would alter BP mechanics a functional movement screening (FMS) was administered to evaluate participants’ mobility and stability within the fundamental patterns related to BP [16].

### 2.4. One Rep Maximum Testing

A week before training began, both groups participated in a fROM 1RM BP testing session. Each participant was instructed on what constitutes the proper form required for a successful 1RM attempt. This form required a pronated hand grip placed within 1.5× the participants’ bi-acromial width which was measured for each participant. Then, while lying supine, the participant must maintain five-point body contact position (back of the head, upper back, glutes, and heel of each foot on the ground) throughout the lift. Progression protocol and obtainment of 1RM was followed as outlined by Haff and Triplett [17] (p. 453).

### 2.5. Exercise Training

Following 1RM testing and prior to training, both groups performed a single familiarization day. While the full range of motion (fROM) group likely did not benefit from this exposure, the pROM group members had no prior experience with the device used to limit ROM. The pROM group used a bar fitted with a commercially available device (Bench Blokz, Bench Blokz, Ruckersville, VA, USA) to limit BP ROM. Intensity for pROM was set at 105% of 1RM. Each week, participants undertook 6 sets for 3 repetitions on day 1 and 7 sets of 3 repetitions on day 2. The Bench Blokz were rotated each week so that the height from bar to sternum would decrease in 1″ increments from 5″ in week 1 down to 2″ in week 4 of training. Participants randomized to fROM increased intensity across the 4 weeks from 80 to 87.5%, making 2.5% jumps one week to the next. Each week, participants undertook 4 sets for 6 repetitions on day 1 and 5 sets of 5 repetitions on day 2. Both groups allowed participants up to 3 min of rest between sets. Training intensities for fROM were determined by assessing appropriate RM for a strength block of training [17] (pp. 459–465) and matched to previously reported [5] and externally valid training loads for pROM training in powerlifters. Training loads were calculated using the equation from Haff [18] of intensity × sets × reps. This resulted in weekly loads of 40.95 in the pROM group and a range of 39.2–42.6 in fROM when both training days were added together. In piloting this approach with individuals of differing arm lengths, total work across individuals was not significantly different.

### 2.6. 3D Videography

Motion capture data was collected using a 12-camera system (Kestral, Motion Analysis, Rohnert Park, CA, USA) sampling at 120 Hz with retro-reflective markers placed on both ends of the bar. Data was tracked using Cortex software (v.7.2 Motion Analysis, Rohnert Park, CA, USA) then exported to a customized MATLAB program version R2024a. Kinematic data was filtered using a 4th-order zero-lag Butterworth filter with an 8 Hz cutoff frequency.

### 2.7. Treatment of Data

Data was analyzed using SPSS v28 (SPSS, Chicago, IL, USA). Treatment of data was 2 × 2 (group × time) ANOVA with repeated measures. For significant interactions, pairwise comparisons with Bonferroni correction were conducted to determine significant changes in dependent variables. A priori significance was set at α < 0.05.

## 3. Results

### Performance Measures

No significant differences existed between groups pre-training. Both groups saw significant increases in 1RM after training (F = 45.82, *p* < 0.001) with no difference between groups. There were no observed differences in average velocity, minimum concentric velocity, 2nd concentric peak velocity, differences between left- and right-hand superior-inferior position during concentric phase, or differences between left- and right-hand height during the concentric phase between groups or pre-to-post training. Multivariate analysis indicated a time effect on 1st concentric peak velocity, mean eccentric velocity, mean concentric force, mean concentric force in quartile 1 and mean concentric force in quartile 2 (Table 2). Pairwise comparisons revealed that full range of motion (fROM) experienced increases in 1st concentric peak velocity (*p* = 0.023) and eccentric velocity (*p* = 0.009), mean concentric force (*p* = 0.04) and mean concentric force in quartile 2 (*p* = 0.01) pre- to post-testing; however, mean concentric force in quartile 1 was not maintained (*p* = 0.13).

## 4. Discussion

Our study was the first to demonstrate progressively increased partial range of motion (pROM) over a 4-week training block conferred similar 1RM improvements to full range of motion (fROM) BP in experienced lifters. We also observed significant differences in eccentric velocity, 1st concentric peak velocity, mean concentric force in quartile 2, and mean concentric force in the fROM group pre- to post-testing.

This study was unique in multiple ways, primarily in its use of progressively increasing pROM over the course of a strength training block. While some studies did change pROM within training days [10,13], others used a set pROM for the duration of the training [15]. After BP training, Massey and colleagues [13] reported significant 1RM increase across all three groups studied: full ROM, partial ROM, and mixed full and partial ROM. After 10 weeks of training at 65% 1RM for 3 sets of 15 reps there were no differences between groups. While our study has results that are, similarly, not significantly different following training, the population and training protocol were not. First, Massey’s pROM group and mixed ROM group did not have a set ROM during training, but rather a range of 2–5″ off the chest they would aim for. Practically, this could be done with the lower intensities utilized in their training but would invite variability in the repetitions. Also, while the lower intensities are appropriate for untrained populations and general exercise recommendation [19], our study utilized well-trained individuals undertaking a discrete strength block [17] (pp. 459–465). However, the similar results indicate that pROM training offers a viable alternative to fROM training in both novice and experienced lifters over short-term periods.

Clark and colleagues’ [10] use of variable ROM over a 12-week training period saw their participants perform five sets per training session, one each of full, three-quarter (3/4), one-half (½), one-quarter (¼), and full ROM in that order. The order was reversed for the second training day each week. They reported no strength changes over the 12-week period to the participants’ 6RM. However, they did report significant improvements to the end-range of motion force output as measured by a bench press throw. We did not see those changes in either group which might be explained by differences in testing. Clark et al. utilized a propulsive throwing form that differed from traditional BP resistance training. In traditional barbell movements, there is a significant breaking phase towards the end of the lift which decreases force and velocity [20]. Perhaps surprisingly, the pROM group in our study did not undergo significant increases in the 3rd or 4th quartiles of the concentric phase even though they had overloaded that range during their training. Our study observed the decrease in force and velocity as expected for a movement with end ROM lockout [20].

The lack of difference in minimum concentric velocity indicates that training did not result in differences regarding a sticking period of the concentric phase. The sticking period has been previously observed to occur in the 30–45% range of the concentric phase [6,14]. Our reporting of increases in 2nd quartile (25–50% ROM) concentric force output in fROM (*p* = 0.01, 15.2 ± 15.3% increase) and pROM (*p* = 0.068, 6.2 ± 10.5% increase), respectively, during 1RM testing indicates that both groups improved performance in this critical range. This appears to be a primary driver of the increase in mean force output in fROM (*p* = 0.04, 6.7 ± 10.4% increase) and pROM (*p* = 0.08, 9.5 ± 5.7% increase), respectively. While the pROM group demonstrated non-significant changes, the positive percent change observed indicates that this group did improve force production through this ROM. Perhaps if the training duration were increased, both training approaches would have resulted in statistically significant increases. The increased velocity of the eccentric phase, paired with the increase in the 2nd quartile concentric force and the increased 1st concentric peak velocity, observed only in the fROM group, suggests that traditional training may better preserve or enhance the efficiency of the stretch-shortening cycle. The limited ROM in the pROM group may have slightly blunted the development of early-phase concentric action compared to the fROM protocol. This difference could result in a better ability to push through the sticking period, yielding improved BP performance. The 1RM testing protocol of the current study neither allowed participants to bounce the bar off their chest nor did it require a pause as seen in competition BP. We did not mandate a competition style pause at the chest for this testing as the participants were not currently trained in that fashion, and we did not want to introduce new variables into their technique which might influence results. It should be noted that the pROM group did see an increase in the 2nd quartile concentric force; however, it is unknown whether significance would have been observed with a longer duration training block or with a larger sample size. Strong stretch shortening cycle performance is highly correlated to early power output in the concentric phase of the bench press [21], which suggests further research should be performed to clear up this question regarding pROM training and eccentric–concentric phase coupling.

Our study was also unique in its use of supramaximal loads during the pROM group’s training block. This loading scheme is appropriate as the nature of pROM training allows for higher-than-normal loads to be lifted [5,9,14]. Ours is the first study to utilize supramaximal fROM loads in the pROM training. Previous studies that had participants undertake training either used submaximal fROM intensities for all pROM groups [15] or did not describe training intensities aside from being work-matched [10]. While it is a limitation of the current study that bar kinematics were not reported at a variety of submaximal attempts on the way to 1RM, there are examples of overloading a portion of ROM with supramaximal intensities in past studies [22]. Doan and colleagues [22] used specialized detachable hooks on the bar so that participants would lower 105% 1RM eccentrically but only concentrically lift 100% 1RM. While cross-sectional, they found that all eight participants increased their 1RM with the increased eccentric load. Without specialized equipment, lifting supramaximal loads presents practical issues for the lifter. While not typically supramaximal, training approaches such as reverse linear periodization (RLP) loading begins by lifting near-maximal loads compared to traditional linear periodization (LP) which begins at moderate loads [23]. However, while both approaches appear to confer strength increases, LP better allows participants to maintain proper form while intensity increases over time [23]. Indeed, Martínez-Cava et al. [15] noted that when fROM BP trained with submaximal intensities (60–80% 1RM), there was carryover in strength to pROMs of ⅓ and ⅔ but not the other direction. However, the bar symmetry variables reported in our study did not find that pROM training with supramaximal loads altered left-to-right hand position either superiorly or inferiorly or in hand height during the concentric phase when compared to submaximal fROM training. This indicates that the current study’s training approach did not induce significant alterations in bar symmetry, allowing participants to maintain form even after training with supramaximal loads. Future studies should endeavor to expand the training duration to determine whether continued progressive or selected pROM supramaximal training alters bar patterns.

This highlights a notable difference in duration between the current study and other training studies. Our study had 4 weeks of training compared to others which were 8–12 weeks [10,13,15]. Future research may focus on elongating the training block by either spending more than 1 week at each of the pROM increments in the current study or loop the training so that after reaching the lowest pROM displacement from the chest participants would begin again at the highest displacement. When adjusting the training duration in future studies, researchers could incorporate mid-point testing to determine whether changes are similar across time. Future studies could also incorporate pROM 1RM testing similar to Martínez-Cava et al. [15] to determine if pROM strength is maintained over a training block duration or if fROM yields superior strength increases in all cases as reported in their study. Clark and colleagues [10] reported that training yielded less strength gains in the second half of the 12-week pre-intervention training period. If it is true that the novelty of pROM training is one of its greatest benefits and with some studies noting that decreased ROM in BP may have better sport specific carryover than fROM BP [9,24], then it may be that longer training blocks yield diminishing returns and a discrete training block of pROM is well suited for off- or pre-season training.

This study had other limitations aside from the lack of reporting submaximal bar kinematics. One such area is small sample size. While 16 participants resulted in a calculated power of 0.845 for the pairwise comparisons, there were null group effects in bar symmetry that may have proven out differently with a larger sample. One challenge to recruiting a larger sample size is the highly trained nature of these participants. In total, our study lasted 6 weeks with 4 weeks of training and disrupting participants’ current training routines without compensation was an obstacle during recruitment. As mentioned, longitudinal training schemes would offer valuable insights into this novel training method, but practical limitations with this population’s training schedules may be prohibitive. The use of a commercially available device to standardize pROM groups offered a solution to common issues with pROM training. Often rolled towels taped together, or wooden boards are used to set the pROM; however, these can deform over time, are challenging to change pROM, or require another person to properly use. The device used in this study is not subject to these issues, thus offering a solution to these training obstacles. Finally, the use of free weights versus smith machines in testing continues to confound results. While smith machines offer a standardized method of pROM training and testing, it may be limiting certain bar movements by forcing symmetries during the exercise. We found no differences between groups’ hand symmetries, though it may be the case that if an individual does not need to focus on combating bar movement, they are better able to put force into the bar, which yields different velocities in a smith machine than would be seen in free weights.

## 5. Conclusions

Our study demonstrates that progressively increasing ROM while maintaining supramaximal loads over a 4-week strength block allows experienced lifters to increase strength comparable to traditional fROM BP training. However, there are notable changes in bar kinematics surrounding the eccentric-concentric phase change that were only seen after fROM BP training. These findings are tempered by the study’s limited sample size and duration, and future studies should examine chronic progressive pROM training with a variety of pROM loading schemes in a variety of populations to determine whether these differences are maintained over time.

## Figures and Tables

**Table 1:** Participant demographics. Mean ± SD.

	n	Age (Years)	Height (cm)	Body Mass (kg)	Body Fat (%)	Years Training
pROM	7	22.3 ± 1.3	180.2 ± 5.2	89.7 ± 7.8	18.34 ± 4.73	6.6 ± 1.2
fROM	9	22.1 ± 1.7	181.3 ± 4.9	90.2 ± 6.5	16.79 ± 4.55	6.2 ± 1.9

**Table 2:** Performance variables. pROM = partial range of motion, fROM = full range of motion. For superior-inferior, a negative value means left is inferior, positive means right is inferior; closer to zero is more symmetrical. For left- and right-hand height, negative means the left hand is higher; closer to zero is more symmetrical. Mean ± SD. * Indicates *p* < 0.05.

	pROM		fROM		Group Effect			Time Effect		
	Pre	Post	Pre	Post	F	Sig.	η^2^	F	Sig.	η^2^
1RM (kg)	107.8 ± 26.9	116.35 ± 26.93	113.93 ± 23.29	121.75 ± 24.14	0.07	0.80	0.57	45.82	<0.001 *	0.77
Mean Concentric Force (N)	1055.85 ± 281.8	1148.42 ± 281.71	1152.39 ± 258.78	1220.54 ± 262.68	0.20	0.66	0.01	8.65	0.011 *	0.38
Quartile 1 Mean Concentric Force (N)	1337.02 ± 353.94	1408.28 ± 318.99	1215.14 ± 342.56	1305.72 ± 319.24	0.07	0.80	0.01	4.66	0.049 *	0.25
Quartile 2 Mean Concentric Force (N)	1164.65 ± 205.88	1239.83 ± 265.87	1027.41 ± 272.23	1156.23 ± 233.64	0.87	0.37	0.06	12.57	0.003 *	0.47
Quartile 3 Mean Concentric Force (N)	1089.55 ± 243.31	1176.43 ± 385.3	1240.71 ± 341.78	1263.94 ± 355.22	0.26	0.62	0.02	0.77	0.39	0.05
Quartile 4 Mean Concentric Force (N)	879.63 ± 289.05	962.73 ± 237.1	878.15 ± 294.54	979.74 ± 287.72	0.07	0.80	0.01	1.37	0.26	0.09
Mean velocity (m/s)	101.46 ± 43.31	115.72 ± 58.17	145.57 ± 53.64	135.62 ± 51.04	0.40	0.54	0.03	0.01	0.91	0.00
1st Concentric Peak Velocity (m/s)	328.06 ± 123.39	338.14 ± 177.96	364.04 ± 122.66	412.09 ± 136.95	1.78	0.20	0.11	4.18	0.047 *	0.23
2nd Concentric Peak Velocity (m/s)	264.54 ± 97.16	278.09 ± 74.19	312.43 ± 90.49	324.95 ± 118.3	0.00	0.99	0.00	0.24	0.64	0.02
Minimum Concentric Velocity (m/s)	41.7 ± 45.4	36.97 ± 70.4	44.97 ± 71.37	35.7 ± 44.44	1.30	0.27	0.09	0.46	0.51	0.03
Eccentric Velocity (m/s)	−469.13 ± 139.45	−525.55 ± 157.48	−464.53 ± 99.99	−566.39 ± 118.3	0.80	0.39	0.05	9.70	0.008 *	0.41
Superior-Inferior Symmetry (mm)	−41.5 ± 91.27	38.17 ± 80.55	−20.14 ± 47.35	−39.17 ± 106.63	3.49	0.08	0.20	1.32	0.27	0.09
Left-Right Symmetry (mm)	−13.00 ± 73.16	29.81 ± 50.71	5.52 ± 80.88	3.56 ± 69.95	1.90	0.19	0.12	1.58	0.23	0.10

## Data Availability

Data may be made available upon request to the corresponding author due to ongoing analysis of related variables.
